# Metabolic Engineering Strategy for *Bacillus subtilis* Producing MK-7

**DOI:** 10.3390/foods14234150

**Published:** 2025-12-03

**Authors:** Shiying Wu, Xiuwen Sun, Tingwen Fan, Fei Lin, Yuan Chi, Huaiyi Yang, Chunhui Zhao

**Affiliations:** 1 Key Laboratory of Plant Biotechnology of Liaoning Province, School of Life Sciences, Liaoning Normal University, Dalian 116081, China; wsymaodou@163.com; 2 State Key Laboratory of Microbial Diversity and Innovative Utilization, Institute of Microbiology, Chinese Academy of Sciences, Beijing 100101, China; sunxw3010@163.com (X.S.); fantw@im.ac.cn (T.F.); linfei23@mails.ucas.ac.cn (F.L.); cy0725933@163.com (Y.C.)

**Keywords:** MK-7, *Bacillus subtilis*, metabolic engineering, biosynthesis

## Abstract

Menaquinone-7 (MK-7) is an important bioactive form of vitamin K_2_ that inhibits vascular calcification, maintains systemic calcium homeostasis, demonstrates high bioavailability, and possesses an extended plasma half-life. *Bacillus subtilis* naturally possesses the complete biosynthetic pathway for MK-7, benefits from a well-characterized genetic background and advanced genome-editing tools, and is regarded as a safe and efficient microbial chassis for industrial MK-7 fermentation. This review summarizes recent advances in the metabolic engineering of *Bacillus subtilis* for high-level MK-7 production, highlights pathway analyses, and discusses engineering strategies targeting four key modules: substrate utilization, secretion, spore/biofilm formation, and oxidative-stress defense.

## 1. Introduction

*Bacillus subtilis*, a species within the genus *Bacillus*, is a motile (possessing flagella), Gram-positive bacterium. It exhibits exceptionally rapid growth rates, remarkable stress resistance, and high adaptability, allowing it to be isolated from diverse environments and to proliferate rapidly under specific conditions. Its metabolic repertoire includes the production of protein coenzymes, hydrolases, vitamins, and more than twenty types of antibiotics [1,2,3].

**Figure 1:**
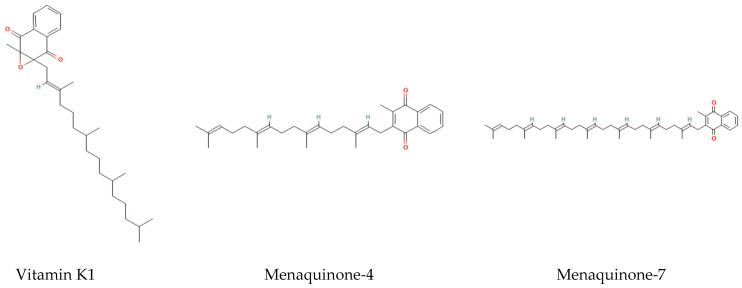
Chemical structures of the major forms of Vitamin K (Vitamin K1, Menaquinone-4, Menaquinone-7).

MK-7 is a form of vitamin K2, belonging to the menaquinones class. Characterized by a side chain containing seven isoprenoid units, MK-7 possesses a longer structure compared to vitamin K1 (phylloquinone) and other K2 forms such as Menaquinone-4 (MK-4) (Figure 1). This extended structure contributes to its longer retention time in the human body and sustains more prolonged plasma concentrations [4,5,6]. MK-4 primarily regulates local tissue calcium balance by reducing the γ-carboxylation of osteocalcin (OC) at the recommended dietary allowance (RDA) level. In contrast, MK-7 promotes the γ-carboxylation of extrahepatic vitamin K-dependent proteins (VKDPs), including osteocalcin (OC) and matrix Gla protein (MGP), thereby contributing to overall calcium balance in the human body. Refs. [7,8,9,10] (differences between these forms are summarized in Table 1).

Intestinal synthesis of vitamin K2 is often limited in children, adolescents, and the elderly due to an unstable gut microbiota, necessitating reliance on exogenous supplementation [11]. Natto represents the richest natural source of MK-7. However, its widespread consumption is hindered by its sticky texture and strong ammonia-like odor, which many find unpalatable. Other fermented foods containing MK-7 (e.g., Gouda cheese) are consumed infrequently in most regions, resulting in inadequate daily intake. Consequently, the industrial production of MK-7 mainly relies on fermenting *Bacillus subtilis*, *Yarrowia lipolytica*, and *Escherichia coli* [12,13].

The chemical synthesis of MK-7 involves multiple reaction steps, requiring heavy metal catalysts and organic solvents. This process risks introducing toxic impurities, entails complex chiral control, necessitates multiple chromatographic purification steps, and ultimately leads to high costs, low yields, significant environmental pollution, and challenges in scaling up [14].

Comparing production hosts reveals distinct advantages for *Bacillus subtilis*. *Yarrowia lipolytica*, as a recognized safe microbial chassis cell, has demonstrated significant advantages in heterologous synthesis of MK-7. It not only has GRAS certification and high food safety, but also has abundant supply of acetyl CoA and a fully functional mevalonic acid pathway, which can efficiently synthesize the pentene side chain of MK-7; Meanwhile, its powerful hydrophobic compound transport and storage capabilities further ensure the accumulation of MK-7. However, the system still requires the external addition of precursor DHNA, which increases production costs; Compared with other efficient hosts, its production capacity still has the potential for further improvement [13]. As a Gram-positive bacterium, *Bacillus subtilis* facilitates easier secretion of products into the extracellular medium. In contrast, *E. coli*, a Gram-negative bacterium with a dual-membrane structure, typically accumulates MK-7 within the inner membrane or periplasmic space. Extracting MK-7 from *E. coli* requires complex cell disruption procedures, increasing both difficulty and cost due to intracellular product retention [15]. From a metabolic engineering perspective, although *E. coli* has mature genetic tools, it lacks the native pathway for MK-7 biosynthesis, necessitating the introduction of heterologous genes. *Bacillus subtilis*, however, possesses an endogenous pathway, making it more amenable to optimization through genetic editing [16]. Regarding safety, most *E. coli* strains harbor endotoxins, requiring complex removal processes. *Bacillus subtilis*, conversely, has Generally Recognized As Safe (GRAS) status, allowing its products to be used directly in dietary supplements [15]. At the same time, using natto as raw material for *Bacillus subtilis* fermentation has three problems: high cost, unstable batch and long time [17,18].

Therefore, current research efforts are predominantly focus on *Bacillus subtilis*. This organism possesses the inherent metabolic pathway for MK-7 synthesis, benefits from a well-characterized genetic background, and has advanced genome editing tools. These attributes facilitate the construction of efficient industrial strains through gene knockout or overexpression of key enzymes, thereby enhancing production efficiency and further increasing MK-7 yield. Fermentation using *Bacillus subtilis* offers significant advantages over traditional chemical synthesis or other microbial fermentation methods, including higher efficiency, superior yield, enhanced safety, and improved process controllability.

Currently, the technology of producing vitamin K_2_ (MK-7) using *Bacillus subtilis* has gradually shifted from traditional natural extraction and chemical synthesis to a strategy combining microbial fermentation and metabolic engineering modification, moving from static modification to dynamic regulation. Although previous studies have increased MK-7 production to several hundred milligrams per liter by enhancing the expression of key enzymes, blocking competitive pathways, and optimizing fermentation conditions, there are still many technical bottlenecks. This review systematically analyzes the key scientific issues in the current production of MK-7 using *Bacillus subtilis*, including insufficient precursor supply, metabolic flux imbalance, and oxidative stress, from five perspectives: optimization of metabolic pathway modules, product secretion mechanisms, regulation of spore and biofilm formation, antioxidant defense systems, and fermentation process synergy. Breaking through the traditional single point genetic modification strategy, innovative synthetic biology dynamic regulation tools and systems biology methods have been introduced, integrating strain modification with fermentation processes in multiple dimensions, and prospectively pointing out fundamental bottlenecks such as unclear secretion mechanisms, providing a clear and forward-looking roadmap for the rational design of the next generation of high-efficiency cell factories.

## 2. Biosynthetic Pathway of MK-7 in *Bacillus subtilis*

**Figure 2:**
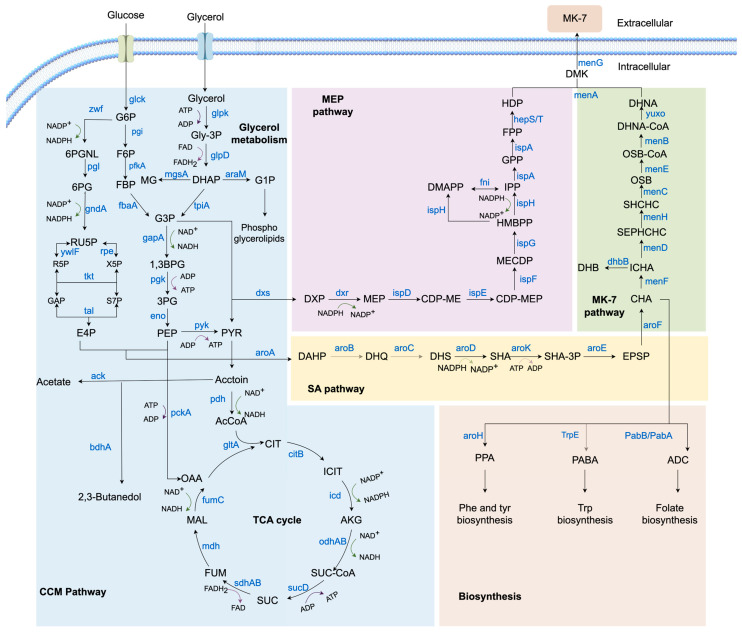
The metabolic pathway of *Bacillus subtilis* producing MK-7 is divided into five modules, namely CCM pathway, SA pathway, MEP pathway, MK-7 pathway and biosynthesis. CCM pathway: GlcK glucose kinase; GlpK glycerol kinase; GlpD glycerol-3-phosphate oxidase; Zwf glucose-6-phosphate1-dehydrogenase; Pgi glucose-6-phosphate isomerase; GndA NADP+-dependent 6-P-gluconate dehydrogenase; YwlF sugar phosphate isomeras; Rpe ribulose-phosphate 3-epimerase; Tkt transketolase; Tal transaldolase; PfkA 6-phosphofructokinase; FbaA fructose-bisphosphate al-dolase; GapA glyceraldehyde-3-phosphate dehydrogenase; Pgk phosphoglycerate kinase; Eno enolase; TpiA tri-osephosphate isomerase; Pyk pyruvate kinase; PckA phosphoenolpyruvate carboxykinase; GltA citrate synthase; CitB aconitate hydratase; Icd isocitrate dehydrogenase; OdhB 2-oxoglutarate dehydrogenase E2 component; SucD suc-cinyl-CoA synthetase alpha subunit; SdhA succinate dehydrogenase flavoprotein subunit; SdhB succinate dehydro-genase iron-sulfur subunit; Mdh malate dehydrogenase; FumC fumarate hydratase; Pdh pyruvate dehydrogenase; AraM glycerol-1-phosphate dehydrogenase; MgsA methylglyoxal synthase; BdhA 2,3-butanediol dehydrogenase; Ack Acetate kinase MEP pathway: Dxs 1-deoxy-D-xylulose-5-phosphate synthase; Dxr 1-deoxy-D-xylulose-5-phosphate reductoisomerase; IspD 2-D-methyl-D-erythritol 4-phosphate cytidylyltransferase; IspE 4-diphosphocytidyl-2C-methyl-D-erythritol kinase; IspF 2-C-methyl-D-erythritol 2,4-cyclodiphosphate synthase; IspG 4-hydroxy-3-methylbut-2-en-1-yl diphosphate synthase; IspH 4-hydroxy-3-methylbut-2-enyl diphosphate reductase;Fni isopentenyl-diphosphate delta-isomerase; IspA farnesyl diphosphate synthase; HepS heptaprenyl diphosphate syn-thase component 1; HepT heptaprenyl diphosphate synthase component 2 MK-7 pathway: MenF menaquinone-specific isochorismate synthase; MenD 2-succinyl-5-enolpyruvyl-6-hydroxy-3-cyclohexene-1-carboxylate synthase; MenH 2-succinyl-6-hydroxy-2,4-cyclohexadiene-1-carboxylate synthase; MenC o-succinylbenzoate synthase; MenE 2-succinylbenzoate-CoA ligase; MenB 1,4-dihydroxy-2-naphthoyl-CoA synthase; YuxO 1,4-dihydroxy-2-naphthoyl-CoA hydrolase; MenA 1,4-dihydroxy-2-naphthoate polyprenyltransferase; MenG deme-thylmenaquinone methyltransferase; SA pathway: AroA 3-deoxy-D-arabino-heptulosonate 7-phosphate synthase; AroB 3-dehydroquinate synthase; AroC 3-dehydroquinate dehydratase; AroD shikimate 5-dehydrogenase; AroE 5-enolpyruvoylshikimate-3-phosphate synthase; AroF chorismate synthase; AroK shikimate kinase Biosynthesis: AroH chorismate mutase; TrpE anthranilate synthase; PabB para-aminobenzoate synthase; PabA anthranilate synthase.

The biosynthetic pathway of *Bacillus subtilis* for MK-7 production consists of four modules: the central carbon metabolic (CCM) pathway, the shikimate (SA) pathway, the methylerythritol 4-phosphate (MEP) pathway, and the MK-7 pathway (Figure 2). Table lists the genes involved in these metabolic pathways. The genes involved in these metabolic pathways are listed in the Supplementary Data.

### 2.1. CCM Pathway

The CCM pathway serves as the core metabolic network of the cell. It is responsible for converting nutrients (such as glucose, glycerol, fatty acids, and amino acids) into energy (e.g., ATP, NADH) and precursor molecules essential for biosynthesis (e.g., acetyl-CoA, α-ketoglutarate). This network comprises four fundamental pathways: glycolysis, gluconeogenesis, the pentose phosphate pathway (PPP), and the tricarboxylic acid (TCA) cycle [19].

In *Bacillus subtilis*, MK-7 biosynthesis relies on two primary carbon sources: glucose and glycerol. A portion of the glucose is channeled through the PPP to generate erythrose-4-phosphate (E4P). This E4P then combines with phosphoenolpyruvate (PEP) derived from glycolysis to enter the SA pathway. Concurrently, another portion of glucose is rapidly metabolized via the Embden-Meyerhof-Parnas (EMP) pathway to generate ATP and NADH. This supports accelerated cellular growth and biomass accumulation during the early fermentation phase.

As glucose becomes depleted, glycerol is metabolized through the glycerol utilization pathway, converting it to glyceraldehyde-3-phosphate (G3P). These C3 metabolites enter the downstream part of the EMP pathway and are eventually converted to pyruvate. Subsequently, G3P and pyruvate, as two direct precursors, jointly enter the MEP pathway to initiate the biosynthesis of isoprene units. Importantly, glycerol utilization is usually inhibited by carbon catabolite repression (CCR) in the presence of glucose, which is relieved after glucose depletion.

Therefore, to enhance MK-7 yield during fermentation, the use of a mixed carbon source (glucose and glycerol) is recommended. This approach balances energy production and precursor availability based on the distinct metabolic properties of each carbon source [20].

#### 2.1.1. Glycerol Metabolism Pathway

Glycerol typically enters the cell via passive transport mediated by the glycerol channel protein encoded by *glpF*. Upon entry, glycerol is phosphorylated to glycerol-3-phosphate (Gly-3-P) by glycerol kinase, encoded by *glpK*, consuming one molecule of adenosine triphosphate (ATP) and generating adenosine diphosphate (ADP). Glycerol kinase is a key enzyme regulating the rate of glycerol metabolism [21,22]. Gly-3-P is subsequently dehydrogenated by glycerol-3-phosphate dehydrogenase (GlpD) to dihydroxyacetone phosphate (DHAP). During this reaction, two electrons are transferred to enzyme-bound flavin adenine dinucleotide (FAD), reducing it to reduced flavine adenine dinucleotide, (FADH_2_). The FADH_2_ then transfers the electrons to the ubiquin in the respiratory chain and reoxidizes itself to FAD [23].

A portion of the DHAP is catalyzed by dihydroxyacetone phosphate mutase, encoded by *araM*, which transfers the phosphate group from the C3 position to C1, forming glycerol-1-phosphate (G1P) [24,25]. Another portion of DHAP is isomerized to G3P by triose phosphate isomerase, encoded by *tpiA*. *tpiA* plays a critical role in balancing subsequent carbon flux, primarily by lowering the activation energy to accelerate the interconversion rate between DHAP and G3P. When *tpiA* expression is aberrant, DHAP accumulates, leading to the formation of toxic methylglyoxal, which inhibits microbial growth.

The resulting G3P is then oxidized to 1,3-bisphosphoglycerate, with the concomitant generation of one molecule of NADH. Phosphoglycerate kinase (Pgk) catalyzes the conversion of 1,3-bisphosphoglycerate to 3-phosphoglycerate, producing one molecule of ATP. Subsequently, 3-Phosphoglycerate is isomerized to 2-phosphoglycerate, which is then dehydrated by enolase (Eno) to form the high-energy compound PEP, priming the system for subsequent ATP generation.

The metabolic pathway from G3P to PEP constitutes a core component of both the CCM pathway and the glycolytic pathway.

#### 2.1.2. EMP Pathway

After glucose enters the cell, it is phosphorylated by glucose kinase (GlcK) to glucose-6-phosphate (G6P). G6P is converted to fructose-6-phosphate (F6P) by phosphoglucose isomerase encoded by the *pgi* gene [26]. Phosphofructokinase (PfkA), the key rate-limiting enzyme in glycolytic flux, phosphorylates F6P to form fructose-1,6-bisphosphate (F1,6BP) [27]. Finally, F1,6BP is cleaved into dihydroxyacetone-phosphate (DHAP) and glyceraldehyde-3-phosphate (GAP) by fructose-bisphosphate aldolase (FbaA). GAP is converted to phosphoenolpyruvate (PEP) and then to pyruvate, which either enters the TCA cycle through acetyl CoA to generate energy or provides carbon for the mevalonic acid pathway, supporting the synthesis of MK-7 isoprenoid side chains [28]. Additionally, PEP serves as a key precursor for DAHP synthesis in the aromatic amino acid pathway [29].

#### 2.1.3. PPP Pathway

G6P is oxidized by glucose-6-phosphate-1-dehydrogenase (Zwf) to 6-phosphoglucono-δ-lactone (6PGNL). Subsequently, 6PGNL undergoes hydrolysis by 6-phosphogluconolactonase (*pgl*-encoded), breaking its cyclic structure to produce the linear compound 6-phosphogluconate (6PG). 6PG is then catalyzed by 6-phosphogluconate dehydrogenase (*gndA*-encoded), which generates ribulose 5-phosphate (Ru5P) along with NADPH.

Ru5P is processed by transketolase (Tkt) and transaldolase (Tal) in the non-oxidative pentose-phosphate pathway, exchanging carbon skeletons with glycolytic intermediates to regenerate GAP and F6P [30]. E4P generated en route exits the PPP and, together with PEP, forms DAHP for aromatic amino-acid biosynthesis.

#### 2.1.4. TCA Cycle

Although the TCA cycle does not directly contribute to the construction of the carbon skeleton of MK-7, it has an important influence on the synthesis efficiency of MK-7 by supplying energy (ATP), reducing equivalents (NADH, FADH_2_) and the replenishment of intermediate metabolites [31,32].

First, the oxidation of acetyl-CoA in the TCA cycle generates abundant reducing equivalents (NADH and FADH_2_), which drive ATP synthesis through oxidative phosphorylation. This supports cellular growth and metabolic activities while providing energy for enzymatic reactions required in MK-7 synthesis. In *Bacillus subtilis*, *citB* encodes aconitate hydratase. The catalytic step of converting citrate to isocitrate (via a cis-aconitate intermediate) represents the second stage of the TCA cycle. This process is subject to coordinated inhibition by glucose and glutamate, serving as a carbon metabolism shutoff mechanism. When readily available carbon sources like glucose are abundant, bacteria prioritize glycolysis for energy production. To conserve energy and resources, the cell downregulates the expression of TCA cycle enzyme expression. “Coordinated inhibition” implies that simultaneous presence of glucose and glutamate exhibits stronger suppressive effects on *citB* expression than their individual presence [33]. Second, in *Bacillus subtilis*, PEP is converted into oxaloacetate (OAA) via phosphoenolpyruvate carboxykinase (PEPCK), encoded by the *pckA*. PEPCK exhibits bidirectional catalytic activity: the forward reaction is glycolytic-driven, converting OAA to PEP [34,35]. When the gluconeogenic pathway is blocked (such as PEPCK deletion or inhibition) or the carbon source is deficient, cells may rely on the reverse reaction of PEPCK as a compensatory mechanism to import the glycolytic intermediate PEP into the TCA cycle.

Additionally, PEPCK serves as an alternative compensatory enzyme to maintain metabolic homeostasis when pyruvate carboxylase becomes inactive [36]. Additionally, *Bacillus subtilis* lacks a complete glyoxylate cycle. Subsequent modifications could introduce isocitrate lyase (AceA) and malate synthase (AceB) from *Escherichia coli* or *Bacillus licheniformis* to minimize carbon loss, enabling more efficient acetyl-CoA production [37]. The glyoxylate cycle enhances an organism’s utilization of acetyl-CoA. With minimal oxaloacetate as a substrate, this cycle can sustainably produce succinate, replenishing four-carbon units (C4 intermediates) for the TCA cycle.

### 2.2. MEP Pathway

The MEP pathway is the core metabolic pathway for *Bacillus subtilis* to synthesize the isoprene side chain precursors (IPP and DMAPP) of MK-7. The formation of DXP is the rate-limiting step in the MEP pathway. Pyruvate and G3P are converted into 1-deoxy-D-xylulose-5-phosphate (Dxp) under the action of 1-deoxy-D-xylulose-5-phosphate synthase (*dxs*-encoding) [38,39]. The second step of MEP involves the reduction and isomerization of DXP, catalyzed by 1-deoxy-D-xylulose-5-phosphate reductoisomerase (Dxr), with NADPH providing reducing power to convert DXP into 2-C-methylerythritol-4-phosphate (MEP) [40]. MEP undergoes two phosphorylation steps under the action of IspD and IspE to form 4-diphosphocytidyl-2-C-methyl-D-erythritol (CDP-ME). Subsequently, CDP-ME cyclizes into the cyclic intermediate ME-CDP, which is then reduced to hydroxymethylbutyryl diphosphate (HMBPP). HMBPP is converted into isopentenyl pyrophosphate (IPP) and dimethylallyl pyrophosphate (DMAPP) in a ratio of approximately 5:1 [41]. The synthesis of MK-7 relies on the continuous supply of IPP and DMAPP, whose intracellular concentrations are strictly regulated by a dual dynamic equilibrium [42]. DMAPP, an isomer of IPP, acts as a “primer” to provide allyl pyrophosphate groups. IPP serves as the direct substrate for terpene elongation, where monomers are sequentially added to the chain’s termini, with each condensation adding five carbon atoms. When DMAPP is insufficient, IDI converts part of IPP into DMAPP to ensure substrate supply. Finally, IPP and DMAPP undergo three consecutive condensation reactions catalyzed by IspA, sequentially forming Geranyl Pyrophosphate (GPP), Farnesyl Pyrophosphate (FPP), and heptanoyl pyrophosphate (HepPP). HepPP provides the C_35_ chain, which condenses with the naphthoquinone skeleton of DHNA to form demethylmenaquinone-7 (DMK-7), which is subsequently methylated to yield the final product, MK-7 [43].

### 2.3. SA Pathway

The SA pathway primarily synthesizes chorismate, the precursor to MK-7’s quinone ring structure. PEP generated through the EMP and E4P from the PPP pathway are condensed into DAHP by DAHP synthase (*aroA*-encoded). Subsequently, DAHP loses a water molecule under the catalysis of DHQ synthase (AroB), forming an unstable intermediate. This intermediate undergoes intramolecular cyclization to form a six-membered ring structure, ultimately yielding 3-dehydroquinate (DHQ). DHQ is then catalyzed by 3-dehydroquinate dehydratase (AroC) to remove a water molecule, producing 3-dehydroshikimate (DHS). AroD-encoded shikimate 5-dehydrogenase reduces the ketone group (C3 position) of DHS to hydroxyl under NADPH’s reducing power, forming shikimate. Shikimate is phosphorylated by AroK to form shikimate-3-phosphate, which transfers its phosphate group to a second molecule of PEP to generate 5-enolpyruvylshikimate-3-phosphate (EPSP). EPSP serves as a precursor for synthesizing aromatic amino acids (phenylalanine, tyrosine, tryptophan), folic acid, and menaquinones. Concurrent studies have demonstrated that glyphosate competitively inhibits the PEP binding site of EPSP synthase, thereby blocking EPSP synthesis [44,45]. Consequently, glyphosate was employed as a chemical regulator to exert pressure by suppressing EPSP synthase, altering carbon metabolic flux. This enabled the strain to survive under 40 μmol/L glyphosate conditions, driving its adaptive evolution in laboratory settings. The evolved strain demonstrated enhanced MK-7 precursor synthesis, ultimately achieving a MK-7 yield of 62 mg/L—2.5 times higher than the original strain—with a production efficiency of 0.42 mg/(L·h), doubling the original strain’s performance [46]. Under chorismate synthase action, EPSP removes the phosphate group at the C_3_ position and proton at the C_6_ position, forming the characteristic enol ether ring structure of chorismate. Concurrently, chorismate exhibit feedback inhibition on EPSP synthase [47].

### 2.4. MK-7 Pathway

The chorismate derived from the SA pathway serve as precursors for the naphthoquinone ring. Under the catalysis of isochorismate synthase (MenF), these chorismate are converted into isochorismate. Subsequent enzymatic actions by 2-succinyl-5-enolpyruvyl-6-hydroxy-3-cyclohexene-1-carboxylate synthase (MenD), MenH, and MenC (OSB synthase) transform them into o-succinylbenzoate (OSB). Notably, MenF and MenD are key genes in DHNA synthesis [48]. OSB undergoes decarboxylation and cyclization catalyzed by MenE and MenB to form 1,4-dihydroxy-2-naphthoate (DHNA). The hydroxyl group of DHNA condenses with the pyrophosphate group of HDP in the MEP pathway, connecting the naphthoquinone ring and side chain to form demethylnaphthoquinone (DMK). Then, DMK undergoes methylation under the action of MenG to form MK-7.

### 2.5. Biosynthesis

In *Bacillus subtilis*, the synthesis of MK-7, aromatic amino acids (phenylalanine, tyrosine, tryptophan), and folate all originate from a common precursor, chorismate. These three pathways diverge into distinct branches after reaching the chorismate node.

Chorismate is converted to prephenate by chorismate mutase, which is then channeled to form phenylalanine and tyrosine. The enzyme anthranilate synthase, encoded by trpE and trpD, catalyzes the conversion of chorismate and glutamine to anthranilate, which undergoes a series of reactions to produce tryptophan.

For folate synthesis, 4-amino-4-deoxychorismate (ADC) synthase, composed of PabB and PabA, first forms ADC from chorismate and glutamine. ADC is then cleaved by ADC lyase to generate para-aminobenzoic acid (PABA). Finally, PABA is assembled with a pteridine moiety to form folate.

## 3. Metabolic Pathway Modification

The metabolic pathway modification of *Bacillus subtilis* typically begins with substrate utilization modules, employing promoters of varying strengths to express key genes or knock out branch pathways to alter metabolic flows. Additionally, modifications are made to secretion systems, spore and biofilm formation modules, and antioxidant defense systems to improve MK-7 secretion efficiency and promote its intracellular accumulation (Table 2).

### 3.1. Substrate Metabolism Module

In the biosynthesis of MK-7 by *Bacillus subtilis*, enhancing precursor supply and suppressing the synthesis of competing byproducts are the most common strategies. By modifying the CCM pathway, we can enhance glycerol and glucose utilization efficiency, improve carbon source utilization efficiency, and provide more precursors and energy for MK-7 synthesis. Using *Bacillus subtilis 168* derivative lacking *dhbB* as the starter strain, competitive pathways were blocked to reduce secondary metabolite diversion, achieving a yield of 55.6 ± 1.2 mg/L. Overexpression of *glpK* and *glpD* further increased glycerol metabolic flux, boosting MK-7 production by 10%. Building on this foundation, knocking out the DHAP competitive metabolic pathway (MG and G1P pathways) directed DHAP into glycolysis, enabling carbon flux enrichment toward the target product to reach 70.3 ± 0.8 mg/L. The methyl ethanedioxyglutarate (MG) pathway involves MgsA catalyzing the conversion of DHAP to MG, while the G1P pathway is mediated by AraM, which reduces DHAP to G1P for phosphoglycerate synthesis [49].

Dxs, Fni, and Dxr are key rate-limiting enzymes in the methylerythritol phosphate (MEP) pathway. To improve MK-7 precursor generation efficiency, studies using wild-type *Bacillus subtilis 168* showed that overexpressing *dxs* under the P_43_ promoter nearly doubled MK-7 production. However, subsequent unilateral overexpression of isopentenyl diphosphate isomerase (*fni*-encoded) under the P_43_ promoter in this background can disrupt terpenoid precursor homeostasis. Without concomitant reinforcement of the downstream menaquinone synthesis module, carbon flux tends to divert towards the isoprenoid branch pathway. This leads to accumulation of the toxic intermediate IPP, inhibiting cell growth and restricting carbon supply for the MK-7 naphthoquinone backbone, ultimately reducing MK-7 yield.

To address this, a combinatorial approach was employed: the native promoters of *dxr* and *menF* were replaced in situ with the P_43_ promoter, and *aroA* was overexpressed under the control of the P_hbs_ promoter. This engineered strain, designated *BS005*, achieved an MK-7 yield of 32.93 mg/L, representing a 2.8-fold increase compared to the base wild-type *Bacillus subtilis 168* strain [50].

MenA serves as a key rate-limiting enzyme that connects DHNA to poly isoprenoid side chains to form DMK. Studies have shown that overexpressing *menA* using P_glgV_ as the promoter in the *ZQ12* starter strain with a yield of 60.40 mg/L increased vitamin K2 production by 2.93 times to 177.38 mg/L. Simultaneously, MenD, located upstream of the vitamin K2 biosynthesis pathway, plays a crucial role in converting 2-ketoglutarate and isochorismate into SEPHCHC—the first rate-limiting step in vitamin K2 biosynthesis, providing the key intermediate SEPHCHC for subsequent synthesis [51]. Using *BSW01* as the starter strain and overexpressing *menD* with P_cspD_ as the promoter alone increased production by 1.75 times to 101.36 mg/L [52].

The 2,3-butanediol dehydrogenase encoded by *bdhA* is responsible for reducing acetoin to 2,3-butanediol, a metabolic byproduct that diverts carbon flux from the synthesis pathways of MK-7 and NK [53]. After knocking out *bdhA*, this metabolic pathway was blocked, leading to a significant reduction in 2,3-butanediol production, with MK-7 output reaching 30.6 mg/L [54].

IPP serves as a critical node in MK-7 synthesis, and insufficient supply of IPP can severely impact MK-7 production. Research has demonstrated that overexpressing genes downstream of the MEP pathway (*ispD*, *ispE*, *ispF*, *ispG*, *ispH*) enhances IPP precursor supply. The baseline strain *BS20* achieved a shake flask yield of 360 mg/L. When further overexpressing *ispD*, the yield increased to 353.2 ± 1.2 mg/L, representing a 10% improvement over *BS20*. Overexpressing *ispF* in *BS20D* yielded 332.6 ± 3 mg/L, showing a 3.9% increase over *BS20* but a 5.8% decrease compared to *BS20D*. Overexpressing *ispE* in *BS20DF* resulted in low glucose consumption and severely inhibited cell growth, necessitating the omission of *ispE*. Instead, overexpressing *ispH* in *BS20DF* achieved a yield of 370.8 ± 5.2 mg/L, representing a 15.8% increase over *BS20*. Finally, overexpressing *ispG* in *BS20DFH* yielded 415 ± 3.2 mg/L, achieving an overall 29.3% increase over *BS20* [55].

### 3.2. Secretion Pathway Module

**Figure 3:**
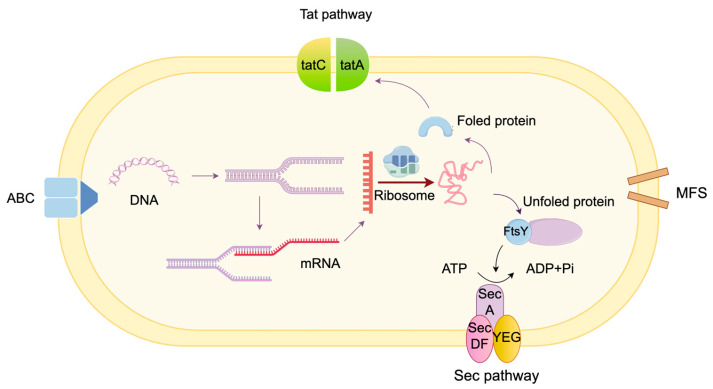
Tat pathway: TatA, TatC, transport folded proteins across the cell membrane; Sec pathway: SecA, SecDF, SecYEG, transport of unfolded proteins, often occurs immediately after translation; FtsY, receptor of SRP (signal recognition particle) pathway; MFS: transport of small molecules through facilitated diffusion or secondary active transport; ABC transporters: participate in the active transport of a variety of small molecule substrates.

MK-7 is a fat-soluble vitamin that is insoluble in water. Approximately half of the MK-7 produced by *Bacillus subtilis* can be secreted extracellularly, as its hydrophobic nature facilitates accumulation at the cell membrane. As shown in Figure 3, its secretion pathways primarily include the Sec pathway, Twin-arginine translocase (Tat) pathway, ABC transporters, and MFS transporters [56]. Transport systems play crucial roles in precursor supply, enzyme localization, and product secretion [57]. The Sec pathway stands as one of the primary protein secretion pathways in *Bacillus subtilis.* The Sec pathway constitutes a complete membrane transport complex comprising the transmembrane protein SecYEG, the paratransporting protein SecDF, and the motor SecA [58]. SecY forms the core subunit of the transmembrane channel, responsible for precursor protein transmembrane transport; SecE stabilizes the structure of SecY; SecG regulates transport efficiency. SecA hydrolyzes ATP to provide energy that drives precursor proteins through the SecYEG channel. SecDF modulates SecA activity while optimizing transport efficiency. The Tat (Twin-arginine translocase) transport system, a crucial double-arginine-dependent transmembrane transporter responsible for folding protein secretion, has been shown to exhibit significant upregulation in the mutant strain BN19-A. Specifically, both *tatA* and *tatC* in the Tat pathway and the *bmr* in ABC transporters showed enhanced expression. This resulted in an elevated extracellular MK-7 ratio to 72.8%, with total MK-7 concentration reaching 22.83 ± 0.52 mg/L—representing a 50.6% increase compared to the parent strain. These observations suggest that the upregulated expression may facilitate MK-7 efflux [59]. Future strategies could involve engineering enhancements to the Tat pathway and ABC transporters to further improve MK-7 production and secretion efficiency.

### 3.3. Spore and Biofilm Formation Module

**Figure 4:**
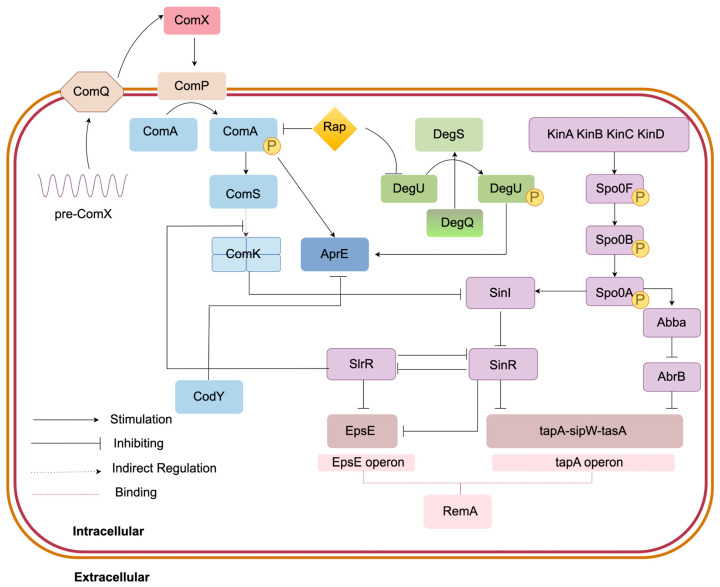
Mechanism of sporulation and biofilm formation in *Bacillus subtilis* producing MK-7. Quorum sensing systems: ComQ, ComX, ComA, ComK, regulate the formation of competent state and the expression of extracellular products;Sporulation module: Spo0A-kinA-E, which senses environmental signals, activates the phosphate cascade, and regulates sporulation; Biofilm forming module: DegU/DegS/DegQ, SinI/SinR, Abba/AbrB, RemA multiple mechanisms jointly control the synthesis of biofilm.

The formation of spores and biofilm during *Bacillus subtilis*’ production of MK-7 primarily relies on the Spo0A-KinA-E regulatory mechanism, SinI/SinR regulation system, AbbA/AbrB regulatory mechanism, as well as the RemA and RemB regulatory mechanisms. Figure 4 demonstrates the specific regulatory processes involved.

#### 3.3.1. Spo0A-KinA-E Regulation Mechanism

In *Bacillus subtilis*, the developmental processes of sporulation and biofilm formation, which impact MK-7 production, are influenced by the ComX quorum-sensing system among other regulatory inputs [60,61]. ComX plays a dual role in *Bacillus subtilis*: it not only inhibits the synthesis of biofilm matrix protein TasA to prevent biofilm formation but also promotes early-stage sporulation [62]. Additionally, ComX induces the expression of AprE, stimulating the production of exoproteases within biofilms, thereby achieving a tripartite regulation system that inhibits biofilm growth, promotes spore generation, and regulates protease activity [63]. When cell density reaches a certain threshold, ComX accumulates and activates the transmembrane histidine kinase ComP, which subsequently activates the phosphorylation cascade through the regulatory factor ComA. Phosphorylated ComA binds to the *sfrA* promoter region, initiating *comS* expression. The ComS protein competitively inhibits the degradation of ComK, thereby increasing ComK concentration. ComK forms tetrameric complexes with its own promoters, triggering positive feedback loops. ComX can promote the formation of the receptive state at low concentration, and promote the formation of spores at high concentration. The activity of ComA is inhibited by Rap proteins.

Sporulation is closely associated with the cellular metabolic state of bacterial cells. Sporulation serves as a survival strategy initiated by *Bacillus subtilis* under nutrient stress conditions such as depletion of carbon or nitrogen sources, but this process terminates metabolic activities. Although spores enhance bacterial survival in extreme environments, the nutrient-deficient feeding conditions in bioreactors may restrict cellular proliferation, making high-density cultivation challenging [63]. *Spo0A*, the primary regulatory gene for sporulation, promotes downstream spore production upon phosphorylation. When environmental stress occurs during the stationary phase, activation of Spo0A (encoded by *spo0A*) causes premature sporulation and inhibits MK-7 synthesis [64]. Therefore, dynamic regulation of Spo0A can block sporulation, prolong the metabolic active phase, and increase MK-7 yield [65]. Additionally, knocking out *kinA*, *kinB*, and *kinE* in the spore phosphorylation pathway prevents phosphate group transfer and reduces Spo0A expression, thereby enhancing MK-7 production. KinC promotes biofilm formation through biphasic regulation of Spo0A activity. In bacterial populations, KinC increases Spo0A activity in fast-growing cells while decreasing it in slow-growing cells. KinC does not simply activate or inhibit Spo0A but optimizes the proportion of biofilm-activating cells through growth-phase-dependent dynamic regulation of Spo0A, thereby enhancing biofilm formation. Additionally, AbrB can inhibit the transcription of Spo0A, thereby delaying sporogenesis [66]. Researchers have developed a dual-function modular quantitative sensing (QS) system based on Phr60-Rap60-Spo0A. By utilizing two native promoters, PabrB and PspoiiA, they modulated gene expression through altering the binding sites of Spo0A-P. This approach successfully increased the production of MK-7 in *Bacillus subtilis 168* from 9 mg/L in shake flasks to 360 mg/L [67].

#### 3.3.2. SinI/SinR Regulatory System

In *Bacillus subtilis*, the thickness of biofilm formation is closely related to the yield of MK-7, while the transition of cells from a planktonic state to a biofilm state is regulated by complex transcriptional mechanisms. The *sin* operon serves as the core regulatory module, directly regulating the expression of genes associated with biofilm formation through antagonistic interactions mediated by its encoded SinR and SinI proteins. The SinR protein encoded by *sinR* belongs to the TetR family of transcription repression proteins, which inhibits the expression of genes related to biofilm formation (tapA-sipW-tasA). The SinI protein encoded by *sinI* acts as an antagonist of SinR, blocking SinR’s DNA binding ability through protein–protein interactions, thereby releasing its inhibition on biofilm-related genes. SinR serves as a global repressor for biofilm genes, while SlrR acts as its antagonist. SlrR binds with SinR to form a heterodimer (SinR_2_-SlrR_2_), which inhibits SinR’s suppression of biofilm genes. Spo0A can also activate the expression of the *sinI* gene, and since SinI is an antagonist of SinR, it regulates SinR expression to modulate biofilm formation [68,69]. Studies have shown that compared to the wild-type *Bacillus subtilis 168* strain, the knockout of *sinR* results in a rougher and drier biofilm with 2.8-fold increased biofilm biomass and 2.6-fold elevated MK-7 concentration, achieving a production yield of 102.56 ± 2.84 mg/L [70]. In *Bacillus subtilis*, a quorum-sensing-based dynamic metabolic regulation system was constructed, significantly enhancing the synthesis efficiency of MK-7. However, traditional static knockout bypassing approaches may cause growth inhibition. To address this issue, a PhrC-RapC-SinR molecular switch was developed, utilizing an auto-triggered gene circuit based on cell density to enable normal expression of growth-phase bypass pathways while efficiently suppressing fermentation phases. Researchers modified the SinR-responsive promoter P_epsA_ to obtain mutant Pe9 with 77.1% inhibition efficiency. By matching different promoter strengths according to the characteristics of various bypass genes, they engineered the *BW7* strain using Pe17 to regulate *alsS-alsD.* This engineered strain achieved an MK-7 yield of 87.52 mg/L, representing a 6.27-fold increase over the original strain, while maintaining unimpeded growth with its OD_600_ consistently exceeding that of the wild-type [71].

**Table 2:** Metabolic engineering of overproduction of *Bacillus subtilis* MK-7.

Target Gene	Method	Strain Background	MK-7 Improvement	MK-7 Titers or Yields	Reference
*glpk*	P_lapS_ promoter	*BSMK*	6%	58.9 ± 1.0 mg/L	[49]
*glpD*	P_lapS_ promoter	*BSMK-1*	10%	61.1 ± 0.5 mg/L	[49]
*mgsA*	Deletion	*BSMK-2*	12%	62.3 ± 0.5 mg/L	[49]
*araM*	Deletion	*BSMK-3*	15%	70.3 ± 0.8 mg/L	[49]
*dxs*, *fni*, *dxr*, *menF*	P_43_ promoter	*Bacillus subtilis168*	2.8	32.93 mg/L	[50]
*aroA*	P_hbs_ promoter				
*menA*	P_glgV_ promoter	*ZQ12*	2.9	177.38 mg/L	[52]
*menD*	P_cspD_ promoter	*BSW01*	1.75	101.36 mg/L	[52]
*bdhA*	Deletion	*Bacillus subtilis 168*	2	30.6 mg/L	[54]
*ispD*	P_43_ promoter	*BS20*	10%	353.2 ± 1.2 mg/L	[55]
*ispF*	P_43_ promoter	*BS20D*	3.9%	332.6 ± 3 mg/L	[55]
*ispH*	P_43_ promoter	*BS20DF*	15.8%	370.8 ± 5.2 mg/L	[55]
*ispG*	P_43_ promoter	*BS20DFH*	29.3%	415 ± 3.2 mg/L	[55]
*spo0A*	P_abrB_ promoter	*Bacillus subtilis 168*	40	360 mg/L	[67]
	P_spoiiA_ promoter				
*sinR*	Deletion	*Bacillus subtilis 168*	2.6	102.56 ± 2.84 mg/L	[70]

#### 3.3.3. AbA/AbrB Regulation Mechanism

AbrB also exerts a certain negative regulatory effect on biofilms, inhibiting the formation of biofilms by suppressing the transcriptional expression of the operon *epsA-O* and *tapA-sipW-tasA* that form the basic matrix of biofilms. AbbA is an antagonist of AbrB, which disrupts the binding of AbrB to DNA by competing for its DNA binding sites [72].

#### 3.3.4. RemA/RemB Regulation Mechanism

The formation of biofilms depends on the synthesis of extracellular polysaccharide (EPS) and amyloid protein (TasA), which are encoded by the eps and *tapA-sipW-tasA* operon, respectively. TasA protein is a key product encoded by the *tapA-sipW-tasA* gene cluster in *Bacillus subtilis.* This protein is cleaved by signal peptidease SipW to form mature TasA, which is then released into the extracellular environment. Its release is associated with sporogenesis and is crucial for maintaining the integrity and stability of the biofilm [73]. RemA can directly bind to the upstream regions of both the *eps* promoter and *tapA* promoter, thereby promoting the expression of biofilm matrix genes. Meanwhile, *sinR* competes with RemA for EPS binding sites. Consequently, SinR can reduce EPS operon expression by blocking RemA binding, thereby decreasing biofilm formation [74,75].

### 3.4. Antioxidant Module

MK-7, a quinone compound, functions as an electron carrier in the cellular respiratory chain, cycling between oxidized (MK-7), semiquinone free radicals (MK-7^−^), and reduced (MK-7H_2_) states. The semiquinone free radicals generate superoxide anions (O_2_^−^) through single-electron reduction of oxygen, initiating reactive oxygen species (ROS) cascade reactions that damage DNA, proteins, and membrane lipids. *Bacillus subtilis*, an aerobic bacterium, contains MK-7 in its cytoplasmic membrane. Since oxygen is crucial for both cell growth and MK-7 accumulation, oxidative stress caused by high MK-7 concentrations remains a key challenge in its production [76,77]. While endogenous scavengers like α-tocopherol (vitamin E) or glutathione (GSH) can directly neutralize ROS, these methods are costly and limited for industrial applications [78]. Altering membrane fatty acid composition (increasing saturated fatty acid ratios) can reduce lipid peroxidation. Secretory systems such as the Tat pathway—through upregulation of *tatAD* and *tatC*—facilitate MK-7 extracellular transport, thereby decreasing intracellular toxicity [54].

Meanwhile, studies have shown that under high oxygen supply conditions, the antioxidant defense system can be activated to protect cells and reduce oxidative damage. At 200 rpm, superoxide dismutase (SOD2) increased by 1.46 times, catalase (CAT) by 2.73 times, alkyl hydroperoxide reductase (AhpF) by 2.42 times, and DNA-binding protein MrgA by 3.3 times. Meanwhile, superoxide dismutase (SOD1) and glutathione peroxidase (GSH-Px) were downregulated. The production of MK-7 reached 18.56 mg/L, doubling that at 100 rpm [65].

## 4. Synergistic Effect of Fermentation Process Optimization and Metabolic Engineering Modification

When engineering microbial strains through metabolic engineering, it is essential to address product synthesis bottlenecks. By optimizing fermentation processes—including medium composition, feeding strategies, and dissolved oxygen control—we can amplify the enhanced metabolic potential, ultimately achieving dual improvements in both yield and efficiency. Optimizing culture media and feeding strategies aligns with the metabolic demands of engineered strains, preventing metabolic imbalances and energy waste.

The control of carbon-to-nitrogen ratio is crucial in the fermentation process of *Bacillus subtilis* producing MK-7. Commonly used carbon sources are glucose and glycerol, while nitrogen sources are mostly cowpea meal and soybean peptone. Although soybean protein hydrolysate serves as a high-performance nitrogen source, it is costly. Through single-factor optimization, the optimal conditions were determined to be 40 °C, pH 7.0, inoculation rate 8%, 200 rpm, and light-shielding conditions, with *Bacillus subtilis 4-b4* isolated from natto, achieving an increase in yield from 2.33 mg/L to 6.12 mg/L. Further supplementation of 20 mg/L of shikimic acid increased MK-7 production to 7.18 mg/L. Through response surface method (RSM), combining optimal parameters with exogenous supplementation achieved a shake flask yield of 7.70 mg/L, 3.31 times higher than initial levels. Finally, in 5 L bioreactors under fed-batch fermentation, MK-7 production reached 23.43 ± 0.41 mg/L after 168 h [79]. Other researchers optimized fermentation conditions for MK-7 production using *Bacillus MM26* isolated from fermented wine through single-factor experiments (OFAT) and RSM. Experiments first screened lactose (6 g/L) and glycine (12 g/L) as optimal carbon/nitrogen sources, determining the optimal pH at 7, temperature at 37 °C, and inoculation rate at 2.5% (2 × 10^7^ CFU/mL). Subsequently, a Box–Behnken design was employed to optimize three factors (cultivation time, lactose concentration, and glycine concentration) at three levels, ultimately achieving a maximum yield of MK-7 at 442 ± 2.08 mg/L, representing a 6.4-fold increase from the initial yield of 67 mg/L [80]. Traditional fermentation methods for MK-7 include solid-state fermentation and stationary-phase liquid fermentation. However, challenges in scale-up persist, including insufficient dissolved oxygen and uncontrollable biofilm formation. While mechanical agitation in liquid fermentation facilitates process amplification, it disrupts biofilm development—a critical factor for MK-7 synthesis [81]. Research has established substrate consumption-product synthesis coupling models to elucidate kinetic patterns of MK-7 fermentation in biofilm reactors, providing theoretical foundations for process scaling-up. These studies also revised the Luedeking–Piret model and confirmed glycerol’s superior metabolic stability as a carbon source [82]. As a non-growth-associated product, MK-7 is primarily synthesized during the stationary phase. During late-stage fermentation when cells enter the senescence phase, MK-7 synthesis may still be enhanced through activation of the respiratory chain. Studies using the Luedeking–Piret equation to model MK-7 accumulation revealed that glycerol is rapidly depleted during the logarithmic growth phase, with cells later relying on nitrogen sources for maintenance. After 24 h of fermentation, supplementing with 10 g/L glycerol resulted in a MK-7 yield of 37.51 mg/L, representing a 1.72-fold increase compared to non-supplemented batches. However, excessive supplementation (up to 15 g/L glycerol) inhibited MK-7 synthesis, likely due to carbon diversion to alternative metabolic pathways [83].

In biofilm formation studies using soy protein hydrolysate (SPHs) as nitrogen source with two-stage aeration fermentation, enhanced biofilm development by *Bacillus subtilis natto* led to a yield of 63.0 ± 2.6 mg/L—a 199.4% increase over the control group, significantly boosting MK-7 production. Two stage aeration fermentation combines the advantages of merged fermentation and biofilm based fermentation, achieving a balance between rapid bacterial growth and efficient MK-7 synthesis by controlling aeration conditions in stages. Firstly, at 37 °C with 150 rpm for 12 h, in an oxygen rich environment, the bacterial cells rapidly proliferate and accumulate a large number of cells, providing sufficient bacterial foundation for subsequent biofilm formation and MK-7 synthesis. The second stage is static fermentation, which is cultured at 37 °C and the bacterial cells gather towards the liquid surface to form a biofilm with a wrinkled structure. Specifically, SPHs induce stronger biofilms with pleated structures, improve cellular activity, delay sporogenesis, and prolong MK-7 synthesis duration. Molecular mechanisms revealed a 0.61-fold reduction in SinR expression, which removes substrate gene repression, while a 0.64-fold decrease in *Spo0A* activity further delays sporogenesis and extends MK-7 synthesis period [84]. Surfactants were added to modulate membrane permeability, enhancing MK-7 secretion rates and overall production. When 20 g/L soybean oil was used as a surfactant, MK-7 yield increased to 40.96 mg/L with a secretion ratio reaching 61.1%, achieving 1.3 times the pretreatment level [85]. Studies have demonstrated that combining soybean protein hydrolysate (SPH) with the surfactant Span 80 (SP80) can enhance MK-7 synthesis efficiency through lifespan extension engineering. In SPH + SP80 medium, both intracellular and extracellular MK-7 yields increased by 5.6-fold and 7.2-fold compared to SP medium, respectively, achieving a total production of 52.9 mg/L with a productivity rate of 0.629 mg/L·h^−1^. Concurrently, the cell mortality rate decreased from 39.5% to 13.5%, while OD_600_ increased by 28% and cell length significantly expanded, indicating synchronized elongation of replication life span (RLS) and chronological life span (CLS) [86].

CaCl_2_·2H_2_O and MgSO_4_·7H_2_O are two commonly used inorganic salt additives that play a key role in promoting bacterial growth and metabolite synthesis. Ca^2+^ helps maintain the integrity of cell membrane structure, enhance membrane permeability to nutrients, promote substrate absorption and metabolite secretion. In *Bacillus subtilis*, Ca^2+^ is also one of the key factors in spore formation, which helps to improve the survival ability and fermentation stability of the bacterial cells. Mg^2+^ is a cofactor for various key enzymes, especially those related to energy metabolism and nucleic acid synthesis. It also helps maintain intracellular charge balance and membrane structure stability. During the fermentation process, CaCl_2_·2H_2_O and MgSO_4_·7H_2_O can be added at a 1:1 ratio. Compared with salt-free fermentation, the yield of MK-7 increased by 165% [87].

In summary, based on different strains obtained and combined with mathematical models, the most suitable parameters should be established to better promote yield growth.

## 5. Challenges and Prospects

*Bacillus subtilis*, a key industrial strain for MK-7 synthesis, has seen significant yield improvements in recent years through metabolic engineering modifications, optimized fermentation parameters, and the application of mathematical modeling in bioreactors [82,88]. However, its industrial-scale adoption still faces multiple bottlenecks, primarily manifested in the following aspects.

### 5.1. The Discharge Mechanism Is Not Clear

Current research on *Bacillus subtilis*’ MK-7 metabolic pathway still lacks sufficient data to substantiate certain conclusions. Many studies only analyze transcriptomic information without molecular-level validation, and most involve iterative modifications without validating individual genes as targeted targets. The functional microdomains (FMMs) embedded in the cell membrane of MK-7 accumulate excessively, leading to decreased membrane fluidity and inhibited cell growth [89]. Current research has only identified four transport systems involved: Sec pathway, Tat pathway, ABC transporter, and MFS transporter. However, studies remain limited in investigating the impact of single genes like *tatAD* and *tatC* on MK-7 production, as well as in constructing high-efficiency transport systems [90].

### 5.2. Insufficient Supply of Precursors

The synthesis pathway of MK-7 involves multiple modules that require precise metabolic flux allocation. However, the current metabolic flux distribution in *Bacillus subtilis* remains imbalanced among these pathways, leading to insufficient supply of certain key intermediates and limiting MK-7 synthesis [91]. IPP is a crucial precursor for MK-7 synthesis. Although *Bacillus subtilis* can synthesize IPP through its endogenous MEP pathway, this pathway may also produce competing metabolites, resulting in flux levels far below the requirements for efficient synthesis. Consequently, IPP supply often becomes a limiting factor. The MVA pathway serves as one of the primary routes for IPP synthesis. Introducing exogenous MVA pathways from *yeast* can enhance IPP supply. However, introducing exogenous genes into *Bacillus subtilis* may also face the challenge of insufficient gene stability.

### 5.3. Limitations of Metabolic Pathway Modification

To overcome the metabolic engineering bottleneck in *Bacillus subtilis* for MK-7 production, researchers have explored various genetic modification strategies in recent years. However, due to the complexity of metabolic pathways and the mutual restriction between various products, obvious limitations have emerged. Overexpression of MEP pathway genes like *dxs* or *ispG* tends to deplete NADPH and ATP, triggering oxidative stress. While enhancing the PPP increases NADPH levels, it simultaneously weakens the EMP pathway, thereby reducing PEP supply. When using strong promoter P_hbs_ to replace the *zwf* promoter in situ, NADPH levels increased by 9.7% compared to baseline, yet MK-7 yield only improved by 11% [50].

### 5.4. Challenges in Applying Bioinformatics Tools

Integrating bioinformatics tools into *Bacillus subtilis* metabolic pathway modification has become one of the more advanced approaches currently available. Most current constraint-based models (CBMs) predict flux under steady-state conditions through metabolic flux analysis (MFA) or flux balance analysis (FBA), which helps reveal biological mechanisms and guide metabolic engineering [92].

The genome-scale metabolic model (GEM) can be analyzed using FBA to integrate *Bacillus subtilis* genomic, transcriptomic, and metabolomic data to simulate metabolic flux distribution under various environmental conditions. This enables rapid identification of critical metabolic nodes, including carbon source allocation and cofactor balance, facilitating precise genetic engineering [93,94]. Additionally, it helps resolve growth-product synthesis conflicts, avoiding metabolic burdens associated with traditional single-gene knockouts or overexpression [95]. Researchers optimized the central carbon pathway using etiBsu1209’s predicted metabolic combinations, achieving an increase in MK-7 yield from 259.7 mg/L to 318.3 mg/L. The model further revealed that the MEP pathway was the primary constraint. Through heterologous expression of *Escherichia coli*’s *dxs* and fusion expression enhancement, MK-7 reached a titer of 451.0 mg/L in shake flasks and 474.0 mg/L in a 50 L bioreactor [96].

FBA is an analytical tool used to simulate the outcomes of a given genetic modification. OptKnock is a design tool that utilizes the FBA framework to automatically search for the optimal gene knockout strategy. Through the joint calculation of FBA and OptKnock, a highly potential modification plan can be quickly obtained, accelerating the development process of engineering strains. A study based on the iYO844 genome-scale metabolic model (GEM) successfully designed an engineered strain *LM01* by single-layer FBA (aimed at maximizing biomass) through knocking out *ackA*, *pta*, *lctE*, and *mmgA* genes. The strain showed significantly higher 2,3-butanediol production compared to the original strain, validating the prediction accuracy [97]. However, when using OptKnock to predict knockouts of *ackA*, *pta*, *mmgA*, and *zwf* genes to form strain *MZ02*, the 2,3-butanediol yield showed little change from the original strain, indicating a failed prediction. In-depth analysis revealed that OptKnock’s error stemmed from its initial flux allocation not considering the lactate pathway during dual-target optimization, mistakenly prioritizing *zwf* gene deletion [97]. Although OptKnock is theoretically perfect, there are indeed many cases in practical applications of *Bacillus subtilis* that have not achieved predictive results. The core assumption of OptKnock is based on the pursuit of maximum growth rate by cells in any environment. But in the actual fermentation process, cells may turn to pursue survival rather than rapid growth. They may lower metabolic rates or redirect resources towards synthesizing protective substances instead of target products. *Bacillus subtilis* has a complex global regulatory network, and knocking out a gene may trigger unknown cascade regulation, thereby inhibiting or activating other unexpected pathways and producing new byproducts. In addition, OptKnock assumes that all reactions can proceed infinitely fast as long as the substrate is sufficient. But in reality, the catalytic efficiency and substrate affinity of enzymes are limited. Model prediction may not be feasible kinetically because the activity of a key enzyme is too low, becoming a bottleneck.

Overall, OptKnock’s failure was mainly due to its failure to integrate theoretical situations with reality. When a prediction is formed, experimenters should not view it as a ‘construction blueprint’, but rather as a ‘preliminary hypothesis to be verified’. Experimenters should conduct further verification based on the actual situation, considering multiple aspects such as growth, energy supply, and byproduct formation. And the actual growth rate, actual by-product secretion rate, and other data obtained from the experiment will be used as constraint conditions in the model, and the metabolic model will be modified with new data to make it more realistic.

FBA assumes that the metabolic network is in a steady state, but in the actual fermentation process, especially during batch fermentation, the concentration of metabolites constantly fluctuates. This may result in deviations in FBA predictions during dynamic processes. At the same time, its prediction results largely depend on the completeness and accuracy of GEM, but significant deviations may occur when the metabolic network is not comprehensive. If a metabolic step is not properly annotated in the genome, resulting in the loss of that pathway in the model. This will prevent FBA from calculating the correct flux through this path, potentially leading to incorrect predictions that the substance cannot be synthesized or degraded. Moreover, many microorganisms possess metabolic pathways that have not yet been discovered by the scientific community. If these paths are missing from the model, FBA naturally cannot simulate them. Using an incomplete GEM for FBA is like planning your itinerary with an incomplete map, so no matter how comprehensive the model prediction is, it needs to be experimentally validated and compared with experimental data for model correction.

Future research could utilize computer simulations to screen optimal modification strategies, reducing trial-and-error experiments and saving time costs. This approach could be applied to Gram-positive bacteria like *Bacillus subtilis* where genetic manipulation is challenging. However, caution should be exercised as some metabolic reactions lack kinetic parameters, leading to discrepancies between FBA predictions and actual conditions. FBA predictions are typically made under ideal cultivation conditions, while industrial fermentation involves substrate fluctuations, oxygen limitation, and byproduct inhibition that cannot be fully accounted for, potentially rendering laboratory optimization strategies ineffective in further production.

## 6. Research Needs

Vitamin K2 has high application value and is difficult to obtain. At present, there are many researchers at home and abroad, mostly through optimizing the medium formula, improving the bioreactor, compound mutagenesis, and enhancing the metabolic flux of vitamin K2 metabolic pathway. *Bacillus subtilis*, as a safe (GRAS), efficient, mature and genetically clear microbial chassis strain, has made significant progress in the production of MK-7 through metabolic engineering. The current research has successfully increased the production of MK-7 from less than 50 mg/L in the early stage to nearly 500 mg/L by enhancing precursor supply, balancing flux distribution, dynamic regulation strategies and other strategies. However, the industrialization of MK-7 still faces multiple bottlenecks. Based on the main knowledge gap to meet the current challenges, we divide the research needs into the following aspects: first, it is urgent to explore the efflux mechanism. At present, although the transport system of MK-7 is preliminarily recognized, it lacks the verification of single gene function, resulting in difficulties in the construction of efficient transport system. Secondly, we should break through the problem of insufficient supply of precursors. To solve the contradiction that the synthetic flux of key precursor IPP is limited due to the competitive pathway, and the introduction of exogenous MVA pathway faces the challenge of gene stability. Moreover, the optimization of metabolic pathway transformation can overcome its inherent limitations, since strengthening a certain pathway often leads to metabolic imbalance and cofactor depletion. Research should focus on the synergy between metabolic engineering and fermentation process to achieve efficient transformation from laboratory to industrial production. Furthermore, a more complete metabolic network should be built. Although bioinformatics tools can accurately predict key targets and greatly improve the efficiency of transformation, their models rely on ideal conditions and ignore the complexity of actual fermentation, which may lead to the prediction results inconsistent with the industrial scale-up effect. Therefore, future breakthroughs need to rely on a deeper analysis of metabolic networks and the development of prediction models that better reflect the actual fermentation environment. Finally, when promoting the industrialization of MK-7 production by *Bacillus subtilis*, the core challenges lie in scalable production, cost-effectiveness and legislative compliance. Solving these problems is the key breakthrough to stimulate interdisciplinary cooperation and lead future research.

This article analyzes the impact of *Bacillus subtilis* on MK-7 production through its metabolic pathway, secretion pathway, spore and biofilm formation module, antioxidant module, and the synergistic effect of fermentation process optimization and metabolic engineering, and summarizes the limitations of current research. Future breakthroughs will rely on in-depth analysis of systems biology, innovation in synthetic biology tools, and optimization of fermentation processes to increase its yield to a new level.

## Figures and Tables

**Table 1:** The difference between the three types of vitamin K.

Type	Source	Half-Life	Function	Metabolic Characteristics	Reference
Vitamin K1	Green leafy vegetables	Short (1–2 h)	Coagulation factor synthesis	Taking up by the liver for clotting	[4,5]
Vitamin K2 MK-4	Animal liver	Middle (2.5 h)	Local tissue calcium regulation	Tachymetabolism	[4,5]
	Egg yolk				
Vitamin K2 MK-7	Natto Fermented foods	Long (68 h)	Systemic calcium metabolism regulation	Slow release	[4,5]

## Data Availability

The original contributions presented in the study are included in the article, further inquiries can be directed to the corresponding author.

## Supplementary Materials

The following supporting information can be downloaded at: https://www.mdpi.com/article/10.3390/foods14234150/s1, Table S1: Regulatory genes for MK-7 biosynthesis in *Bacillus subtilis*.
