# Supplementary material for: Metabolic Engineering Strategy for Bacillus subtilis Producing MK-7
Source: Foods. 2025 Dec 3;14(23):4150. doi: 10.3390/foods14234150 (PMC12692220; doi:10.3390/foods14234150)
Supplement: Supplementary file 1 [file foods-14-04150-s001.zip › foods-4000856-supplementary.pdf]

Supplementary material for "Foods"

Metabolic engineering strategy for Bacillus subtilis producing MK-7

Shiying Wu [12], Xiuwen Sun[2],Tingwen Fan[2],Fei Lin[2],Yuan Chi[2], Huaiyi Yang[2],* , Chunhui Zhao[1],*

1 School of Life Sciences, Liaoning Normal University; Key Laboratory of Plant Biotechnology of Liaoning Province, Dalian, Liaoning 116081, China; wsymaodou@163.com (S.W.)

2 State Key Laboratory of Microbial Diversity and Innovative Utilization, Institute of Microbiology, Chinese Academy of Sciences, Beijing 100101, China; sunxw3010@163.com(X.S.);; fantw@im.ac.cn(T.F.）; cy0725933@163.com(Y.C.)；linfei23@mails.ucas.ac.cn(F.L.)

***Correspondence: yanghy@im.ac.cn(H.Y.)；zch@lnnu.edu.cn(C.Z.)**

Summary

1. **Tables**

   Table 1. Regulatory genes for MK-7 biosynthesis in Bacillus subtilis.

Table 1. Regulatory genes for MK-7 biosynthesis in *Bacillus subtilis*.

Gene	Function	Optimum pH	Optimum Temperature（℃）	Km Value（μM）	Cofactor	EC Number
glck	glucose kinase	7.5	32		ATP	EC 2.7.1.2
glpK	glycerol kinase					EC 2.7.1.30
glpD	glycerol-3-phosphate oxidase				FAD	EC 1.1.3.21
zwf	glucose-6-phosphate 1-dehydrogenase	9.2	25			EC 1.1.1.49
pgl	6-phosphogluconolactonase					EC 3.1.1.31
pgi	glucose-6-phosphate isomerase					EC 5.3.1.9
gndA	NADP+-dependent 6-P-gluconate dehydrogenase				NADP+	EC 1.1.1.44
ywlf	sugar phosphate isomeras					EC 5.3.1.6
rpe	ribulose-phosphate 3-epimerase					EC 5.1.3.1
tkt	transketolase					EC 2.2.1.1
tal	transaldolase					EC 2.2.1.2
pfkA	6-phosphofructokinase					EC 2.7.1.11

fbaA	fructose-bisphosphate aldolase	7.5		2		EC 4.1.2.13
gapA	glyceraldehyde-3-phosphate dehydrogenase		0.1 - 1		NAD+	EC 1.2.1.12
pgk	phosphoglycerate kinase					EC 2.7.2.3
eno	enolase					EC 4.2.1.11
tpiA	triosephosphate isomerase					EC 5.3.1.1
pyk	pyruvate kinase				ADP	EC 2.7.1.40
pckA	phosphoenolpyruvate carboxykinase				ATP	EC 4.1.1.49
gltA	citrate synthase					EC 2.3.3.1
citB	aconitate hydratase	7.5	25		iron-sulfur centre	EC 4.2.1.3
icd	isocitrate dehydrogenase		25	0.0274		EC 1.1.1.42
odhB	2-oxoglutarate dehydrogenase E2 component					EC 2.3.1.61
sucD	succinyl-CoA synthetase alpha subunit					EC 6.2.1.5
sdhA	succinate dehydrogenase flavoprotein subunit	7.4			FAD	EC 1.3.5.1
sdhB	succinate dehydrogenase iron-sulfur subunit	7.4			FAD	EC 1.3.5.1
mdh	malate dehydrogenase			0.1-0.14	NAD+、NADH	EC 1.1.1.37
fumC	fumarate hydratase					EC 4.2.1.2
pdh	pyruvate dehydrogenase					EC 1.2.4.1
araM	glycerol-1-phosphate dehydrogenase					EC 1.1.1.261
mgsA	methylglyoxal synthase					EC 4.2.3.3
bdhA	2,3-butanediol dehydrogenase					EC 1.1.1.4
ack	Acetate kinase					EC 2.7.2.1
dxs	1-deoxy-D-xylulose-5-phosphate synthase					EC 2.2.1.7

dxr	1-deoxy-D-xylulose-5-phosphate reductoisomerase					EC 1.1.1.267
ispD	2-D-methyl-D-erythritol 4-phosphate cytidylyltransferase	8	30	0.1248		EC 2.7.7.60
ispE	4-diphosphocytidyl-2C-methyl-D-erythritol kinase				ATP	EC 2.7.1.148
ispF	2-C-methyl-D-erythritol 2,4-cyclodiphosphate synthase					EC 4.6.1.12
ispG	4-hydroxy-3-methylbut-2-en-1-yl diphosphate synthase					EC 1.17.7.1
ispH	4-hydroxy-3-methylbut-2-enyl diphosphate reductase					EC 1.17.7.4
fni	isopentenyl-diphosphate delta-isomerase	6.5 - 7	37		NADH、NADPH	EC 5.3.3.2
ispA	farnesyl diphosphate synthase					EC 2.5.1.1
hepS	heptaprenyl diphosphate synthase component 1	7.5 - 9				EC 2.5.1.30
hepT	heptaprenyl diphosphate synthase component 2	7.5 - 9				EC 2.5.1.30
menF	menaquinone-specific isochorismate synthase					EC 5.4.4.2
menD	2-succinyl-5-enolpyruvyl-6-hydroxy-3-cyclohexene-1-carboxylate synthase			0.0077 - 0.514	thiamine diphosphate	EC 2.2.1.9
menH	2-succinyl-6-hydroxy-2,4-cyclohexadiene-1-carboxylate synthase					EC 4.2.99.20
menC	o-succinylbenzoate synthase					EC 4.2.1.113
menE	2-succinylbenzoate-CoA ligase	7.5	34	0.044 - 0.88	ATP	EC:6.2.1.26

menB	1,4-dihydroxy-2-naphthoyl-CoA synthase					EC 4.1.3.36
yuxo	1,4-dihydroxy-2-naphthoyl-CoA hydrolase					EC 3.1.2.28
menA	1,4-dihydroxy-2-naphthoate polyprenyltransferase					EC 2.5.1.74
menG	demethylmenaquinone methyltransferase					EC 2.1.1.163
aroA	3-deoxy-D-arabino-heptulosonate 7-phosphate synthase	9		1.4 - 1.76		EC 2.5.1.54
aroB	3-dehydroquinate synthase			0.055	NAD+	EC 4.2.3.4
aroC	3-dehydroquinate dehydratase			0.1071		EC 4.2.1.10
aroD	shikimate 5-dehydrogenase					EC 1.1.1.25
aroE	5-enolpyruvoylshikimate-3-phosphate synthase			0.16 - 0.43		EC 2.5.1.19
aroF	chorismate synthase				NADPH	EC 4.2.3.5
aroK	shikimate kinase					EC 2.7.1.71
aroH	chorismate mutase	7.5	37	0.067 - 16		EC 5.4.99.5
trpE	anthranilate synthase					EC 4.1.3.27
pabB	para-aminobenzoate synthase					EC 2.6.1.85
pabA	anthranilate synthase					EC 2.6.1.85

* Data from Brenda enzyme database.
